# Improved Oral Health Is Associated with a Lower Risk of Late Onset Ankylosing Spondylitis: A Nationwide Cohort Study

**DOI:** 10.3390/jcm13061606

**Published:** 2024-03-11

**Authors:** Min Kyung Chung, Yoonkyung Chang, Jung-Hyun Park, Gwang Hyun Leem, Tae-Jin Song

**Affiliations:** 1 Division of Rheumatology, Department of Internal Medicine, Ewha Womans University College of Medicine, Seoul 07804, Republic of Korea; 2 Department of Neurology, Mokdong Hospital, Ewha Womans University College of Medicine, Seoul 07985, Republic of Korea; ykchang@ewha.ac.kr; 3 Department of Oral and Maxillofacial Surgery, Mokdong Hospital, Ewha Womans University College of Medicine, Seoul 07985, Republic of Korea; omspark07@ewha.ac.kr; 4 Department of Neurology, Seoul Hospital, Ewha Womans University College of Medicine, Seoul 07804, Republic of Korea; shalomlkh@gmail.com

**Keywords:** periodontitis, oral hygiene, tooth brushing, ankylosing spondylitis, focal disease, source of infection

## Abstract

**Background**: The aim of this study was to evaluate the association of oral health status and habits with the occurrence of ankylosing spondylitis (AS) in a nationwide population-based cohort in a longitudinal setting. **Methods:** A total of 2,415,963 individuals aged 40–79 years who underwent oral health examinations were included from the National Health Insurance Service-National Health Screening (NHIS-HEALS) cohort of Korea between 2003 and 2004. The occurrence of AS was analyzed according to the oral health status and oral hygiene habits. **Results:** Among 2,271,221 of the participants, AS occurred in 6366 (0.3%) participants over 16.7 years. The likelihood of AS was higher in participants who had periodontitis (hazard ratio [HR]: 1.33, 95% confidence interval [CI]: 1.20–1.46, *p* < 0.0001) and more missing teeth (HR: 1.68, 95% CI: 1.42–1.99, *p* < 0.0001). However, better oral hygiene habits such as frequent tooth brushing (HR: 0.77, 95% CI: 0.71–0.83, *p* < 0.0001) and a history of dental scaling within the last year (HR 0.88, 95% CI 0.82–0.95, *p* = 0.001) were associated with a lower occurrence of AS. **Conclusions:** Periodontitis and an increased number of missing teeth could be related to the occurrence of late-onset AS. Improved oral hygiene care may attenuate the likelihood of late-onset AS.

## 1. Introduction

Ankylosing spondylitis (AS) is a chronic inflammatory disease characterized by axial and enthesis inflammation leading to new bone formation [1]. AS frequently occurs during the third decade of life, most commonly among young people, with 0.1–1.4% of all people prevalence the disease [2]. Although AS is usually an early-onset disease, and given that the assessment of SpondyloArthritis International Society (ASAS) classification criteria defines inflammatory back pain as starting before the age of 45 years, late-onset AS increases with a higher life expectancy [3,4].

Periodontitis is one of the most common infectious diseases and is characterized by chronic inflammation of the tooth-supporting tissue causing bone loss [5]. Although periodontitis originates from the local inflammatory process associated with the accumulation of oral bacteria in dental plaque, severe damage to soft and hard periodontal tissues occurs as systemic inflammation continues owing to immune system activation [6]. Thus, periodontitis can influence various systemic diseases including diabetes, certain cancers, neurodegenerative diseases, osteoporosis, coronary artery disease, and strokes [7]. Systemic inflammation and elevated pro-inflammatory cytokines in chronic periodontitis can be associated with insulin resistance leading to diabetes and with endothelial dysfunction leading to impaired vasodilation and promoting atherosclerosis [8]. Recent studies have shown that cytokines produced in periodontitis are involved in the regulation of osteoclasts and there is a link between periodontitis and systemic bone loss such as osteoporosis [9]. Periodontitis has also been shown to be associated with some systemic inflammatory diseases such as rheumatoid arthritis (RA), systemic lupus erythematosus (SLE), and inflammatory bowel diseases (IBD) [10,11,12]. Numerous clinical, epidemiologic, and serologic studies have demonstrated a notable association between RA and periodontitis [13,14]. Low-grade inflammation related to SLE is correlated with the dysbiosis of oral microbiota in periodontal diseases [11]. Moreover, interleukin (IL)-17, which has a pivotal role in many immune-mediated inflammatory diseases including psoriasis, inflammatory arthritis, and AS, has been suggested as a potent proinflammatory mediator that can explain the occurrence of comorbid periodontitis [12].

Evidence has been reported that periodontitis can affect and be affected by systemic inflammation and that it can be a modifiable risk factor in the systemic inflammatory burden [15]. Most commonly, two dimensions are used to stage and classify periodontitis: severity, defined by clinical attachment loss, radiographic bone loss, and tooth loss; and complexity, defined by probing depth and marginal bone loss. However, grading that predicts the progression of periodontitis, including age, smoking, diabetes, and inflammatory markers, is also emerging, reflecting the influence of systemic inflammation of periodontitis [16].

Several studies have suggested an association between AS and periodontitis [17,18,19]. A previous study reported that patients with AS have 6.8 times the chance of periodontitis [17] and 1.8 times the chance of having a previous diagnosis of chronic periodontitis [18]. A systemic review on periodontitis and its association with AS showed significantly higher prevalence of periodontitis in patients with AS compared to patients without AS [20]. A study that evaluated periodontal measurements in patients with AS and healthy controls also identified the intercorrelation between periodontitis and AS. Periodontal indicators such as bleeding on probing (BOP) and clinical attachment loss (CAL) showed a significant positive correlation with Bath Ankylosing Spondylitis Metrology Index (BASMI), an indicator of motor limitation in AS, and with Maastricht Ankylosing Spondylitis Enthesitis Score (MASES), which reflects disease activity of AS by the extent of enthesis involvement [21]. Conversely, another study showed that chronic periodontitis was associated with the severity of spinal immobility but not with AS [19]. This inconsistency might arise from small study sample sizes or inaccurate case definition of each disease, such as that resulting from self-reporting. Until now, there have been limited longitudinal studies on the relationship of overall oral health or related habits such as tooth loss, tooth brushing, and dental scaling, with AS in the general population.

In the current study, we hypothesized that poor oral health status, such as periodontitis, is associated with an increased risk of AS, and that improved oral hygiene habits are related to a lower risk of AS. This study aimed to investigate the association of oral health examination findings and habits with the occurrence of AS using a longitudinal database from a nationwide population-based cohort.

## 2. Materials and Methods

### 2.1. Data Source

This research used the National Health Insurance Service-National Health Screening (NHIS-HEALS) cohort database of Korea. The NHIS, as Korea’s exclusive insurance provider, serves nearly 97% of Koreans, while the Medical Aid program, also operated by the NHIS, serves the remaining 3% of the population [22]. The NHIS encourages its subscribers to undergo annual health screening that follows standardized protocols. The NHIS-HEALS cohort database comprises demographic and socioeconomic data, health screening results, and a claims database that encompasses details such as diagnosis, prescription, and treatment methods. In addition to measuring height, weight, blood pressure, and performing laboratory tests and lifestyle questionnaires relating to oral hygiene habits, the health screening process also involves a dental examination conducted by dentists. Participants underwent dental evaluation conducted by dentists to assess the number of teeth lost and other dental issues. The individuals in the NHIS-HEALS dataset did not participate in the planning, analysis, or documentation of this study. The Institutional Review Board of Ewha Woman’s University College of Medicine approved this study (2020-08-018, date of approval: 1 September 2020) and waived the requirement for participant consent.

### 2.2. Study Population

**Figure 1:**
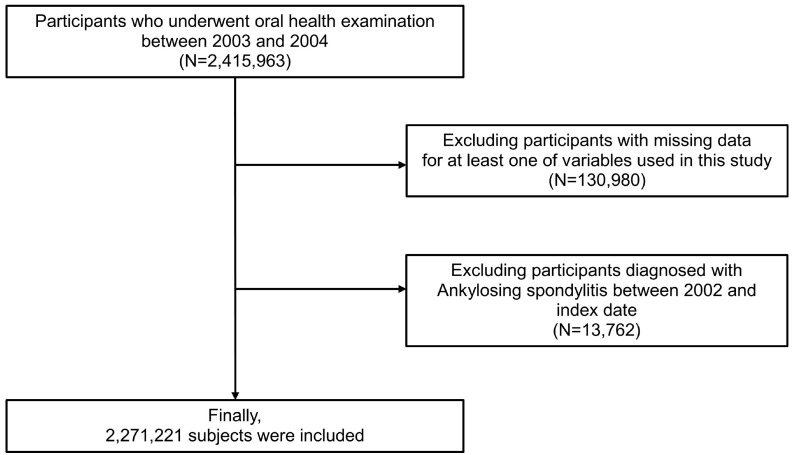
Flow chart of study population.

The NHIS-HEALS cohort comprises representative, randomly selected samples of approximately 2,415,963 individuals aged 40–79 years who participated in health screenings between 2003 and 2004, approximately 40% of the total population who received national health examinations (dataset number: NHIS-2022-01-313) [22,23]. Participants with at least one missing key variable (*n* = 130,980) were excluded. Then, participants with a previous diagnosis of AS between 2002 and the index date of oral health examination (*n* = 13,762) were excluded to provide 1 year washout. Finally, 2,271,221 participants were included for the analysis (Figure 1).

### 2.3. Definition and Variables

The index date was set as the date when the oral health examination was performed. The baseline characteristics of the participants, including age, sex, household income, and body mass index (BMI), were collected at the index date. Data regarding smoking behavior, frequency of alcohol intake (per week), and regularity of physical exercise (measured by frequency per week) were gathered through the questionnaires. Smoking status was categorized as non, former, and current smokers. Periodontitis was defined as meeting one of the two conditions: (1) having at least two claims for periodontitis diagnosis codes (International Classification of Diseases, 10th Revision (ICD)-10 codes: K052-054), with at least one recorded claim under any of following procedure codes: U0010, U1010, U2211, U2221-22, U2231-33, U2240, U4454-55, U1051-52, U1060, U4660, U4662, or M0111); or (2) the presence of a periodontal pocket as detected by a dentist on a dental examination. A probing depth of 4 mm or more was defined as a pathological periodontal pocket on the examination [24]. The dentists also detected the number of teeth missing during the oral health examination. We classified the number of missing teeth into the following four groups: 0, 1–7, 8–14, and more than 15, irrespective of underlying causes such as periodontal disease or other dental problems. The participants’ oral hygiene habits were categorized based on three factors: frequency of tooth brushing (0–1 time, 2 times, and ≥3 times per day), whether they visited a dental clinic for any reason, and whether they underwent dental scaling at least once in the past year. Comorbidities were detected between January 2002 and the index date via the following criteria based on ICD-10 diagnostic codes (Appendix A) [25,26].

### 2.4. Study Outcomes

The study outcome was the occurrence of AS, defined as at least one claim of diagnostic code ICD-10 M45, with an individual copayment beneficiaries program (ICBP) code (V140). Since the Korean government subsidized medical expenses for patients with rare and intractable diseases (RID) through an ICBP, AS was designated as an RID covered by this program. All patients enrolled as having AS were required to have their diagnosis certified by physicians following the modified New York criteria for AS [27] with reasonable test results, which makes the AS diagnostic code reliable. The follow-up period was from the index date until the occurrence of AS, the participant died, or the end of December 2020—whichever appeared first.

### 2.5. Statistical Analysis

The groups’ baseline characteristics were analyzed using the chi-square test for categorical variables and the independent *t*-test for continuous variables. Continuous variables are demonstrated as the means ± standard deviation, and categorical variables are presented as numbers (percentages). Propensity score matching (PSM) was applied to equalize the baseline characteristics and reduce potential confounding. PSM was conducted using a greedy nearest-neighbor algorithm with a 1:4 ratio. A standardized mean difference (SMD) < 0.1 indicated suitability. We calculated the AS incidence by dividing the total number of AS cases by the cumulative persons-per-years. Kaplan–Meier survival curves with the log-rank test was employed to assess the association of oral health status and oral hygiene habits with incident AS risk. We used Cox’s proportional hazard regression to determine the risk of oral health status and oral hygiene habits for the occurrence of AS and calculated the hazard ratio (HR) and 95% confidence interval (CI). The multivariable regression model was created with adjusting age, sex, body mass index, income, alcohol consumption, smoking status, frequency of physical activity, and comorbidities (hypertension, diabetes mellitus, dyslipidemia, atrial fibrillation, cancer, renal disease, RA, and SLE). All statistical analyses were performed using the Statistical Analysis System software (SAS version 9.2, SAS Institute, Cary, NC, USA). All values with *p*-values < 0.05 were considered statistically significant.

## 3. Results

The average age of the 2,271,221 included participants was 42.1 ± 12.8 years, and 66.3% were male. Among all participants, 922,356 (40.6%) brushed their teeth over three times a day, 13,841 (0.6%) had more than 15 missing teeth, and 523,203 (23.0%) had received dental scaling within the last year. The baseline characteristics and comparative analyses based on the presence of periodontitis are demonstrated in Table 1. The results of PSM applied to equalize the baseline characteristics and reduce potential confounding were demonstrated in Table 2.

**Figure 2:**
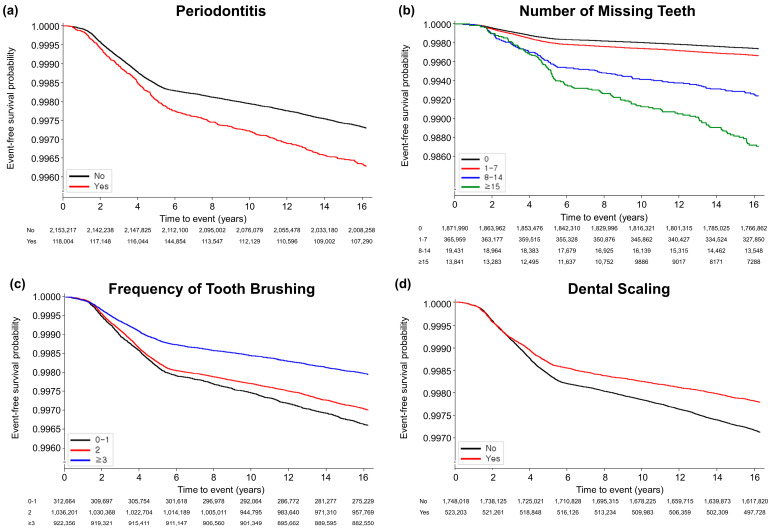
Kaplan-Meier survival curves for occurrence of ankylosing spondylitis according to oral hygiene status and habits. (**a**) Periodontitis (*p* < 0.0001); (**b**) number of missing teeth (*p* < 0.0001); (**c**) frequency of tooth brushing (times/per day) (*p* < 0.0001); (**d**) dental scaling within previous year (*p* = 0.001).

A total of 6366 (0.3%) participants developed AS over the median duration of 16.7 [interquartile range 16.2–17.2] years. Figure 2 illustrates Kaplan–Meier survival curves for AS according to the oral health conditions and oral hygiene habits. The curve demonstrated that the participants with periodontitis and increased number of missing teeth exhibited a greater risk for AS (*p* < 0.0001 for both), and improved oral hygiene habits such as more frequent tooth brushing and having received dental scaling within the past year were also related, with a lower occurrence of AS (*p* < 0.001).

In multivariable analysis, periodontitis was associated with the higher occurrence of AS (adjusted HR: 1.33, 95% CI: 1.20–1.46, *p* < 0.0001). An increased number of missing teeth was correlated with an increased risk for the occurrence of AS. The adjusted HRs (in reference to the subject without missing teeth) were: 1.68 (95% CI: 1.42–1.99, *p* < 0.0001, *p* for trend <0.0001) for subjects with more than 15 missing teeth. Moreover, brushing teeth more frequently showed negative correlation with the occurrence of AS. Compared to the subjects who brushed their teeth less than once a day, those who brushed over three times a day (adjusted HR: 0.77, 95% CI: 0.71–0.83, *p* < 0.0001) had a decreased risk of AS. Furthermore, those who underwent dental scaling within one year showed a reduced risk for the occurrence of AS (adjusted HR: 0.88, 95% CI: 0.82–0.95, *p* = 0.001) (Table 3).

According to the cox regression analysis for each variable, the risk of AS was higher in those aged 65–79 years than in those aged 40–64 years (adjusted HR: 3.23, 95% CI: 2.85–3.65, *p* < 0.0001). Drinking alcohol more than five times a week increased the HR of AS to 1.38 (1.10–1.72) according to the crude model, but did not show significance after adjustment. Moreover, having metabolic comorbidities such as hypertension and diabetes mellitus were associated with increased risk of AS (adjusted HR: 1.21, 95% CI: 1.09–1.33, *p* = 0.001 and adjusted HR: 1.21, 95% CI: 1.05–1.39, *p* = 0.009). Overall, the HR of AS occurrence related to oral health status and behaviors maintained a similar pattern even after PSM and variable adjustment (Supplementary Table S1).

## 4. Discussion

This study demonstrated that periodontitis and increased tooth loss are associated with an increased risk of AS occurrence and that improved oral hygiene through frequent tooth brushing and dental scaling was related to a decreased risk of AS.

Several studies have investigated the association between AS and periodontitis [17,18,19,20,28,29,30]. A German study comparing patients with AS and healthy controls showed that patients with AS had a higher risk of periodontitis than the controls [17]. Additionally, it has been reported that bleeding on probing among the periodontal parameters was significantly higher in patients with AS than in the control group [28]. In a study using Taiwanese administrative claim data, patients with AS had an increased history of having been previously diagnosed with periodontitis than the control group, suggesting a bidirectional association between the two diseases [18]. Moreover, some previous studies have suggested a relationship between immobility and periodontitis in patients with AS [19,29]. Systemic reviews on the association between periodontitis and the disease activity of AS have shown that patients with AS have a high prevalence of periodontitis, which is related to poor oral hygiene [20,30]. However, other studies have failed to show an association between periodontitis and AS. Although bleeding on probing was significantly associated with AS and spondylarthritis, some studies have shown that other periodontal parameters and periodontitis were not associated with AS [19,28,31]. The differences in the results of previous studies could be attributed to the difference in the definition of the disease group, a small sample size, or a short follow-up period. In this research, periodontitis was associated with an increased AS risk. This result is considered to be reliable given that errors due to disease definition were minimized as we identified periodontitis not by questionnaires but according to the result of direct oral examination by a dentist or diagnostic code combined with dental procedures, and AS according to the accurate diagnostic code registered as RID in the NHIS. This finding indirectly suggests that treating and controlling periodontitis may contribute to reducing risk factors for developing AS. However, the mechanism for the high risk of AS occurrence in patients with periodontitis is unclear, and research related to this mechanism is still limited. IL-17, which plays a vital role in the development and ossification of AS, has also been revealed as important in periodontitis. Additionally, periodontitis is associated with spondyloarthropathy other than AS, such as psoriasis and IBD, meaning that additional research will be needed.

In addition, a distinctive aspect of this research is that we mainly identified late-onset AS in patients with periodontitis because the participants who underwent oral health examinations were aged 40–79 years. AS usually occurs in young men aged 20–40, and it seldom occurs as a late-onset disease known to have slightly different epidemiologic and clinical characteristics. Late-onset AS shows lower human leukocyte antigen B27 (HLA-B27) positivity, higher levels of inflammatory markers, more cervical or peripheral joint involvement, and a higher incidence in females [32,33,34]. Therefore, these differential characteristics of late-onset AS might have influenced the result as we mainly detected late-onset AS in the study.

In this study, an increased number of missing teeth was related to a higher risk of AS. In a previous study examining the association between tooth loss and AS disease parameters, older age and increased BASMI were correlated with a lower number of remaining teeth in patients with AS [35]. Additionally, the prevalence of loose teeth or loss of natural teeth was increased in patients with AS compared to the non-AS group [36]. Although tooth loss is a complex problem with various causes, it can be considered as a periodontal disease burden. Hence, our results align with the close relationship between periodontitis and AS. Moreover, as tooth loss is related to nutrient intake, it may contribute to the development of AS by contributing to malnutrition [37] or even dysbiosis of the gut microbiome [38].

Contrary to the association of periodontitis with AS observed in this study, behaviors that improve oral hygiene such as tooth brushing or dental scaling were related to a reduced risk of AS. Until now, no previous study has investigated the effect of oral hygiene improvement on the risk of AS. D’Aluto et al. showed decreased C-reactive protein and inflammatory marker levels after periodontal therapy [39], while other studies revealed that frequent tooth brushing effectively prevented several systemic diseases [25,26,40]. The mechanism by which improvement in oral hygiene reduces the risk of developing AS is unclear. AS develops as a combination of various genetic and environmental factors. Among the known environmental factors, infection is acknowledged to play a contributing role as both germ-free HLA-B27 transgenic rats and SKG mice are disease-free [41,42]. Moreover, a systematic review of the relationship between periodontal pathogens and AS suggested that HLA-B27-specific cytotoxic T-cell mediated autoimmune responses can be initiated when HLA-B27-positive individuals are infected with *Porphyromonas gingivalis* which has argine- and lysine-specific protease, and peptidylarginine deiminase (PAD) [43]. Therefore, tooth brushing and dental scaling, both of which reduce this bacterial load, may be factors that hinder autoimmune responses, leading to a decreased risk of AS.

This study has some limitations that warrant discussion. First, the degree and severity of periodontitis, including detailed attachment loss, could not be investigated because of the nature of the data. However, it was possible to evaluate the presence of periodontitis relatively accurately given that the experts directly inspected and evaluated the participants’ oral condition. Second, periodontitis on dental examination was defined as having a periodontal pocket with a probing depth of four millimeters or more. Since the American Academy of Periodontology advocates considering both clinical attachment loss and probing depth in periodontitis [44] and the Community Periodontal Index classifies shallow pockets as greater than four millimeters and deep pockets as exceeding six millimeters, milder forms of periodontitis might be included in this study. Third, due to the nature of insurance claim data, data related to the HLA-B27 positivity rate and AS disease activity were not included. Therefore, the possibility that it included the onset at the time and the delayed diagnosis cannot be ruled out. However, habits that improve oral health have also been shown to be associated with a lower incidence risk of AS, raising the possibility of a relationship. Lastly, given this study design, a causational relationship between oral health status, habits, and the risk of AS could not be established.

Nevertheless, this study has some strengths in that it identified the risk of incident patients of AS according to the presence of periodontitis, which might imply a bidirectional effect between periodontitis and AS. Moreover, it is meaningful in that it suggests that changes in oral hygiene habits, modifiable factors, are related to the occurrence of AS. Most importantly, this study has long tracked large-scale, representative national data, which improves the accuracy of our result, showing the association between oral health and the occurrence of late-onset AS.

Further prospective studies are required to uncover the impact of periodontitis and oral hygiene habits on the occurrence of AS. Future research, including the occurrence of AS with serial periodontal status, the effect of local periodontal treatment on the disease activity of AS, the impact of periodontitis on AS at a young age, and experimental studies mediating the correlation, might be needed to elucidate the association between oral health and AS.

## 5. Conclusions

The presence of periodontitis and an increased number of missing teeth could be associated factors for developing late-onset AS. In contrast, improved oral hygiene care, including frequent tooth brushing and dental scaling, might be related to a reduced risk for late-onset AS.

## Figures and Tables

**Table 1:** Baseline characteristics of participants according to periodontitis.

Variable	Total	Periodontitis (−)	Periodontitis (+)	*p*-Value
Number of participants (%)	2,271,221 (100.0)	2,153,217	118,004	<0.0001
Age, years	42.14 ± 12.75	42.08 ± 12.72	43.29 ± 13.32	<0.0001
<65	2,136,898 (94.1)	2,028,391 (94.2)	108,507 (92.0)	
≥65	134,323 (5.9)	124,826 (5.7)	9497 (8.0)	
Sex				<0.0001
Male	1,505,305 (66.3)	1,422,811 (66.1)	82,494 (69.9)	
Female	765,916 (33.7)	730,406 (33.9)	35,510 (30.1)	
Body mass index (kg/m^2^)	23.54 ± 14.22	23.53 ± 14.19	23.68 ± 14.75	<0.0001
Household income				<0.0001
Q1, lowest	590,312 (26.0)	560,074 (26.0)	30,238 (25.6)	
Q2	821,367 (36.2)	778,034 (36.1)	43,333 (36.7)	
Q3	597,235 (26.3)	565,897 (26.3)	31,338 (26.6)	
Q4, highest	262,307 (11.6)	249,212 (11.6)	13,095 (11.1)	
Smoking status				<0.0001
Never	1,273,168 (56.1)	1,208,682 (56.1)	64,486 (54.6)	
Former	244,362 (10.8)	230,468 (10.7)	13,894 (11.8)	
Current	753,691 (33.2)	714,067 (33.2)	39,624 (33.6)	
Alcohol consumption (days/week)				<0.0001
None	1,518,140 (66.8)	1,440,863 (66.9)	77,277 (65.5)	
1–4	694,720 (30.6)	657,789 (30.6)	36,931 (31.3)	
≥5	58,361 (2.6)	54,565 (2.5)	3796 (3.2)	
Regular physical activity (days/week)				<0.0001
None	1,183,012 (52.1)	1,121,610 (52.1)	61,402 (52.0)	
1–4	926,030 (40.8)	878,599 (40.8)	47,431 (40.2)	
≥5	162,179 (7.1)	153,008 (7.1)	9171 (7.8)	
Comorbidities				
Hypertension	656,672 (28.9)	620,352 (28.8)	36,320 (30.8)	<0.0001
Diabetes mellitus	156,574 (6.9)	146,373 (6.8)	10,201 (8.6)	<0.0001
Dyslipidemia	292,308 (12.9)	276,226 (12.8)	16,082 (13.6)	<0.0001
Atrial fibrillation	8513 (0.4)	7816 (0.4)	697 (0.6)	<0.0001
Cancer	21,733 (1.0)	19,799 (0.9)	1934 (1.6)	<0.0001
Renal disease	21,010 (0.9)	19,443 (0.9)	1567 (1.3)	<0.0001
Rheumatoid arthritis	7982 (0.4)	7325 (0.3)	657 (0.6)	<0.0001
Systemic lupus erythematosus	1415 (0.1)	1312 (0.1)	103 (0.1)	0.001
Oral hygiene behaviors				
Frequency of tooth brushing (times/day)				<0.0001
0–1	312,664 (13.8)	295,522 (13.7)	17,142 (14.5)	
2	1,036,201 (45.6)	981,304 (45.6)	54,897 (46.5)	
≥3	922,356 (40.6)	876,391 (40.7)	45,965 (39.0)	
Number of missing teeth				<0.0001
0	1,871,990 (82.4)	1,776,314 (82.5)	95,676 (81.1)	
1–7	365,959 (16.1)	345,952 (16.1)	20,007 (17.0)	
8–14	19,431 (0.9)	18,037 (0.8)	1394 (1.2)	
≥15	13,841 (0.6)	12,914 (0.6)	927 (0.7)	
Dental visit for any reason				<0.0001
No	1,360,869 (59.9)	1,292,701 (60.0)	68,168 (57.8)	
Yes	910,352 (40.1)	860,516 (40.0)	49,836 (42.2)	
Dental Scaling				<0.0001
No	1,748,018 (80.0)	1,658,214 (77.0)	89,804 (76.1)	
Yes	523,203 (23.0)	495,003 (23.0)	28,200 (23.9)	

*p*-value by Chi-square test. Data are expressed as mean ± standard deviation, or *n* (%). SMD: standardized mean difference; Q: quartile.

**Table 2:** Baseline characteristics of participants according to periodontitis before and after propensity score matching.

Variable	Before PSM (*n* = 2,271,221)			After PSM (*n* = 590,014)		SMD
	Periodontitis (−)	Periodontitis (+)	*p*-Value	Periodontitis (−)	Periodontitis (+)	
Number of participants (%)	2,153,217	118,004	<0.0001	472,010	118,004	
Age, years	42.08 ± 12.72	43.29 ± 13.32	<0.0001	43.12 ± 13.28	43.29 ± 13.32	0.001
<65	2,028,391 (94.2)	108,507 (92.0)		433,836 (91.9)	108,507 (92.0)	
≥65	124,826 (5.7)	9497 (8.0)		38,174 (80.1)	9497 (8.1)	
Sex			<0.0001			−0.014
Male	1,422,811 (66.1)	82,494 (69.9)		332,514 (70.5)	82,494 (69.9)	
Female	730,406 (33.9)	35,510 (30.1)		139,496 (29.5)	35,510 (30.1)	
Body mass index (kg/m^2^)	23.53 ± 14.19	23.68 ± 14.75	<0.0001	23.6 ± 18.3	23.68 ± 14.75	0.003
Household income			<0.0001			0.007
Q1, lowest	560,074 (26.0)	30,238 (25.6)		122,565 (26.0)	30,238 (25.6)	
Q2	778,034 (36.1)	43,333 (36.7)		172,197 (36.5)	43,333 (36.7)	
Q3	565,897 (26.3)	31,338 (26.6)		125,149 (26.5)	31,338 (26.6)	
Q4, highest	249,212 (11.6)	13,095 (11.1)		52,099 (11.0)	13,095 (11.1)	
Smoking status			<0.0001			−0.002
Never	1208,682 (56.1)	64,486 (54.7)		257,923 (54.6)	64,486 (54.6)	
Former	230,468 (10.7)	13,894 (11.8)		52,614 (11.2)	13,894 (11.8)	
Current	714,067 (33.2)	39,624 (33.6)		161,473 (34.2)	39,624 (33.6)	
Alcohol consumption (days/week)			<0.0001			0.003
None	1,440,863 (66.9)	77,277 (65.5)		308,396 (65.3)	77,277 (65.5)	
1–4	657,789 (30.6)	36,931 (31.3)		149,401 (31.7)	36,931 (31.3)	
≥5	54,565 (2.5)	3796 (3.2)		14,213 (3.0)	3796 (3.2)	
Regular physical activity (days/week)			<0.0001			0.009
None	1,121,610 (52.1)	61,402 (52.0)		245,126 (51.9)	61,402 (52.0)	
1–4	878,599 (40.8)	47,431 (40.2)		193,179 (40.9)	47,431 (40.2)	
≥5	153,008 (7.1)	9171 (7.8)		33,705 (7.1)	9171 (7.8)	
Comorbidities						
Hypertension	620,352 (28.8)	36,320 (30.8)	<0.0001	140,890 (29.9)	36,320 (30.8)	−0.014
Diabetes mellitus	146,373 (6.8)	10,201 (8.6)	<0.0001	40,291 (8.5)	10,201 (8.6)	−0.015
Dyslipidemia	276,226 (12.8)	16,082 (13.6)	<0.0001	61,914 (13.1)	16,082 (13.6)	−0.033
Atrial fibrillation	7816 (0.4)	697 (0.6)	<0.0001	2788 (0.6)	697 (0.6)	−0.004
Cancer	19,799 (0.9)	1934 (1.6)	<0.0001	7827 (1.7)	1934 (1.6)	0.002
Renal disease	19,443 (0.9)	1567 (1.3)	<0.0001	6239 (1.3)	1567 (1.3)	−0.004
Rheumatoid arthritis	7325 (0.3)	657 (0.6)	<0.0001	2699 (0.6)	657 (0.6)	−0.001
Systemic lupus erythematosus	1312 (0.1)	103 (0.1)	0.001	413 (0.1)	103 (0.1)	0.001
Oral hygiene behaviors						
Frequency of tooth brushing (times/day)			<0.0001			−0.010
0–1	295,522 (13.7)	17,142 (14.5)		67,291 (14.3)	17,142 (14.5)	
2	981,304 (45.6)	54,897 (46.5)		219,403 (46.5)	54,897 (46.5)	
≥3	876,391 (40.7)	45,965 (39.0)		185,316 (39.3)	45,965 (39.0)	
Number of missing teeth			<0.0001			0.021
0	1,776,314 (82.5)	95,676 (81.1)		385,229 (81.6)	95,676 (81.1)	
1–7	345,952 (16.1)	20,007 (17.0)		78,160 (16.6)	20,007 (16.9)	
8–14	18,037 (0.8)	1394 (1.2)		4787 (1.0)	1394 (1.2)	
≥15	12,914 (0.6)	927 (0.8)		3834 (0.8)	927 (0.8)	
Dental visit for any reason			<0.0001			0.001
No	1,292,701 (60.0)	68,168 (57.8)		274,845 (58.2)	68,168 (57.8)	
Yes	860,516 (40.0)	49,836 (42.2)		197,165 (41.8)	49,836 (42.2)	
Dental Scaling			<0.0001			−0.005
No	1,658,214 (77.0)	89,804 (76.1)		360,699 (76.4)	89,804 (76.1)	
Yes	495,003 (23.0)	28,200 (23.9)		111,311 (23.6)	28,200 (23.9)	

*p*-value by Chi-square test. Data are expressed as mean ± standard deviation, or *n* (%). PSM matching ratio: 1:4. SMD: Standardized mean difference; Q: quartile.

**Table 3:** Association of oral health status and oral hygiene behaviors with occurrence of ankylosing spondylitis.

	Number of Participants	Number of Events	Event Rate (%)(95% CI)	Person-Years	Incidence Rate(per 1000 Person-Years)	Adjusted HR(95% CI)	*p*-Value
Oral health status							
Periodontitis							
No	2,153,217	5912	0.27 (0.27, 0.28)	35,309,467.51	0.17	1 (reference)	
Yes	118,004	454	0.38 (0.35, 0.42)	1,898,712.29	0.24	1.33 (1.20, 1.46)	<0.0001
Number of missing teeth							
0	1,871,990	4862	0.26 (0.25, 0.27)	30,838,486.07	0.16	1 (reference)	
1–7	365,959	1215	0.33 (0.31, 0.35)	5,904,907.22	0.21	1.12 (1.05, 1.19)	0.001
8–14	19,431	140	0.72 (0.60, 0.84)	284,109.54	0.49	1.44 (1.22, 1.72)	<0.0001
≥15	13,841	149	1.08 (0.90, 1.25)	180,676.98	0.82	1.68 (1.42, 1.99)	<0.0001
Oral hygiene behaviors							
Frequency of tooth brushing (times/day)							
0–1	312,664	1118	0.36 (0.34, 0.38)	5,019,097.36	0.22	1 (reference)	
2	1,036,201	3261	0.31 (0.30, 0.33)	16,953,586.96	0.19	0.99 (0.92, 1.06)	0.683
≥3	922,356	1987	0.22 (0.21, 0.22)	15,235,495.48	0.13	0.77 (0.71, 0.83)	<0.0001
Dental visit for any reason							
No	1,360,869	3977	0.29 (0.28, 0.30)	22,237,856.02	0.18	1 (reference)	
Yes	910,352	2389	0.26 (0.25, 0.27)	14,970,323.78	0.16	0.98 (0.93, 1.04)	0.485
Dental scaling							
No	1,748,018	5183	0.30 (0.29, 0.30)	28,562,069.05	0.18	1 (reference)	
Yes	523,203	1183	0.23 (0.21, 0.24)	8,646,110.75	0.14	0.88 (0.82, 0.95)	0.001

Multivariable model was adjusted with sex, age, body mass index, income levels, smoking, alcohol consumption, regular physical activity, hypertension, diabetes mellitus, dyslipidemia, atrial fibrillation, cancer, renal disease, rheumatoid arthritis, and systemic lupus erythematosus. CI: confidence interval; HR: hazard ratio.

## Data Availability

The data used in this study are available from the National Health Insurance Service-National Health Screening Cohort (NHIS-HEALS) database, but restrictions apply to the public availability of these data used under license for the current study. Requests for access to the NHIS data can be made through the National Health Insurance Sharing Service homepage [http://nhiss.nhis.or.kr/bd/ab/bdaba021eng.do], accessed on 15 September 2020. For access to the database, a completed application form, research proposal, and application for approval from the institutional review board should be submitted to the inquiry committee of research support in the NHIS for review.

## Supplementary Materials

The following supporting information can be downloaded at: https://www.mdpi.com/article/10.3390/jcm13061606/s1, Supplementary Table S1: Cox regression model result of Ankylosing spondylitis.

## Abbreviations

AS	Ankylosing spondylitis
ASAS	Assessment of SpondyloArthritis International Society
BASMI	Bath Ankylosing Spondylitis Metrology Index
BMI	Body mass index
BOP	Bleeding on probing
CAL	Clinical attachment loss
CI	Confidence interval
HEALS	Health screening
HLA-B27	Human Leukocyte Antigen B27
HR	Hazard ratio
IBD	Inflammatory bowel disease
ICBP	Individual copayment beneficiaries program
ICD	International classification of diseases
IL	Interleukin
MASES	Maastricht Ankylosing Spondylitis Enthesitis Score
NHIS	National Health Insurance Service
PAD	PeptidylArginine Deiminase
PSM	Propensity score matching
RA	Rheumatoid arthritis
RID	Rare and intractable diseases
SLE	Systemic lupus erythematosus
SMD	Standardized mean difference

## Appendix A

Hypertension was defined as satisfying one of the following criteria: (1) at least one claim of diagnostic codes ICD-10 I10–15, with the prescription of an antihypertensive agent; (2) two or more claims of diagnostic codes ICD-10 E11–14; (3) systolic/diastolic blood pressure ≥140/90 mmHg; or (4) self-reported hypertension in the questionnaire. Diabetes mellitus was defined as satisfying one of the following criteria: (1) at least one claim of diagnostic codes ICD-10 E11–14 with the prescription of an antidiabetic agent; (2) two or more claims of diagnostic codes ICD-10 E11–14; (3) fasting serum glucose level ≥7.0 mmol/L; or (4) self-reported diabetes mellitus in the questionnaire. Dyslipidemia was defined as satisfying one of following criteria: (1) at least one claim of diagnostic codes ICD-10 E78 with the prescription of a dyslipidemia-related agent; (2) two or more claims of diagnostic codes ICD-10 E78; or (3) total cholesterol ≥240 mg/dL. Atrial fibrillation was defined as two or more claims of diagnostic code ICD-10 I48. Cancer was defined as having one admission or at least three outpatient claims of diagnostic code ICD-10 C00–97, with a specific registration code of ‘V027’ or ‘V193–4’. Renal disease was defined as two or more claims of diagnostic codes ICD-10 N17-19, I12-13, E082, E102, E112, and E132, or an estimated glomerular filtration rate <60 mL/min/1.73 m^2^. Rheumatoid arthritis was defined as two or more claims of diagnostic codes ICD-10 M05 pr specific registration code of ‘V223’. Systemic lupus erythematosus was defined as two or more claims of diagnostic codes ICD-10 M32 or specific registration code of ‘V136’ [25,26].
